# Ameliorative effect of black tea extract on the skin of D-galactose-induced aging mice

**DOI:** 10.3389/fnut.2023.1275199

**Published:** 2023-09-15

**Authors:** Xiaojie Zheng, Wenbin Deng, Xinzhou Wang, Zufang Wu, Chong Li, Xin Zhang

**Affiliations:** ^1^Southern Zhejiang Key Laboratory of Crop Breeding, Wenzhou Vocational College of Science and Technology (Wenzhou Academy of Agricultural Sciences), Wenzhou, Zhejiang, China; ^2^Zhejiang Tiefengtang Health Technology Co., Ltd., Wenzhou, China; ^3^Department of Food Science and Engineering, Ningbo University, Ningbo, China; ^4^Graduate School, Xuzhou Medical University, Xuzhou, China

**Keywords:** black tea extract, skin aging, anti-aging, biochemical index, tissue structure

## Abstract

Aging is a universal and irreversible process, and the skin is an important feature that reflects the aging of the organism. Skin aging has been a focus of attention in recent years because it leads to changes in an individual’s external features and the loss of many important biological functions. This experiment investigated the improvement effect of black tea extract (BTE) on the skin of aging mice under D-galactose induction. After 6 weeks of administration, the changes in skin bio-chemical indices and tissue structure were compared with the blank and positive control groups. It was observed that BTE increased water and hyaluronic acid (HA) content, decreased malondialdehyde (MDA) content, enhanced superoxide dismutase (SOD), glutathione peroxidase (GSH-Px), and catalase (CAT) activities in the skin of aging mice, and improved the structure of aging damaged skin tissues and increased the content of total collagen. The experimental results showed that BTE can play a significant anti-aging effect on the skin, which can be used as a functional food for aging inhibition.

## Introduction

Aging is a universal process that began at the origin of life about 3.5 billion years ago. Various harmful substances in the body’s tissues and organs accumulate gradually with age, causing damage to their corresponding functions and eventually leading to death. Factors that contribute to aging include development, genetic defects, living environment, and disease (1). Humans have a long history of exploration of aging. In the 19th century, researchers began to investigate the nature of aging by experimental methods, and several aging theories were published within a few years. Among them, the free radical aging theory proposed by the American scientist Denham Harman in 1954 is the most representative, which proposes that “the reactive free radicals generated in the organism react with intracellular components to trigger changes associated with aging” (2). The body has its function of scavenging free radicals, but as we age, this function degrades, and excess free radicals oxidize unsaturated fatty acids in cell membranes, disrupting the cell membrane structure and generating harmful oxidation products that trigger the aging of the body (3, 4).

Aging of the skin is the most visual manifestation of the aging of the organism. Skin aging is a multi-systemic degenerative process involving the skin and skin support systems (5). Many factors contribute to skin aging, which can be mainly summarized as external and internal factors. Skin aging caused by external factors refers to skin damage caused by environmental issues such as prolonged sun exposure and exposure to airborne pollutants, which can be effectively prevented by applying protection (6, 7). Internal factors refer to skin aging caused by heredity, also known as natural aging or intrinsic aging. As we age, the accumulation of harmful products in the skin as well as the loss of water and collagen can lead to symptoms such as atrophy, dryness, thinning of the thickness of the skin, and loss of elasticity, which are the main reasons for the appearance of wrinkles (8). As the largest organ in the human body, aging not only affects the appearance of the skin, but also destroys its function. The skin has many functions, including barrier, thermoregulation, metabolism, and immunity, and the proper functioning of these functions is a key factor in maintaining human health. The aging of skin tissues due to aging disrupts their normal functions, and therefore older adults are more susceptible to skin diseases such as psoriasis and eczema than younger adults (9–11). In addition, aging skin also means that outside pathogens can more easily break through the immune barrier established by the skin to invade the body, which in turn can cause various diseases. It becomes important to know how to reduce the health risks of aging skin.

Black tea is a fully fermented tea made from fresh leaves through withering, kneading, fermentation, and drying. Human beings have a long history of drinking black tea. According to records, black tea was first discovered in China around the 15th century and was introduced to Europe in the early 16th century, and drinking black tea soon became a popular fashion in Europe and gradually became popular worldwide (12). Past research has confirmed that regular consumption of black tea can have a variety of beneficial health effects, including cardiovascular protection and metabolic boost (13). The origin of black tea is distributed all over the world, and the main places of origin include China and India (14). According to reports, black tea accounts for 78% of the world’s total tea consumption, making it the most popular tea category (15).

Black tea extract refers to using black tea as raw material, extraction, and separation by physical or chemical methods, based on not changing the structure of its original components, directed to obtain one or more of the active ingredients. Most of the common BTE on the market today is obtained by hot water extraction, which retains most of the nutrients of black tea and ensures the safety of the product, mainly including catechins, theaflavins and caffeine, and other phenolic substances, and these products are often sold as health products (15, 16). Oxidative damage is the main cause of skin aging, and the large amount of phenolics contained in BTE provides an extremely strong antioxidant base (17). In past studies, the antioxidant capacity of BTE has been verified in both *in vitro* and animal experiments (18, 19). Therefore, it is not difficult to speculate that BTE can also provide a better anti-aging effect on the skin.

With socio-economic development, the need for skin care is expanding for all age groups. Outside of skin care products, people are looking to their diets in the hope that by adjusting their diets they can achieve the effect of slowing down the aging of their skin. Black tea is produced all over the world and has a large production and consumer base, which makes it very commercially viable. BTE is extracted from black tea, retaining most of its bioactive components, and is now widely used in weight-loss and blood pressure-lowering products (20, 21). Compared to BTE’s other functional products, there is still more room for exploration in the efficacy of slowing down the aging process. Until now, research on BTE has focused on its antioxidant function, and the ability of BTE to inhibit skin aging is not clear, with a lack of corresponding research data. Therefore, this topic takes BTE as the research object, establishes a D-galactose-induced aging animal model, and explores the inhibitory effect of BTE on skin aging by measuring the skin-related physicochemical indexes as well as observing the stained sections of skin tissues, which implied the potential of BTE to enhance skin quality and prevent aging.

## Materials and methods

### Experimental material

BTE is provided by Taiyi Health Industry Co., Ltd. (Ningbo, China), which is a red-dish-brown powder made from black tea by hot water extraction, filtration and lyophilization. Our preliminary experiments explored 20.92% catechin and 5.75% theaflavin content in BTE. Vitamin C was purchased from Sinopharm Chemical Reagent Co., Ltd. (Beijing, China), and used as a positive control. Elisa kits for SOD, GSH-Px, CAT, MDA, HA, and total collagen were purchased from KAIB Bio-technology Co., Ltd. (Shanghai, China). HE and immunohistochemistry staining kits were purchased from Xavier Bio-technology Co., Ltd. (Wuhan, China). All the reagents used in the experiment were analytically pure.

### Experimental animal

SPF grade male KM mice (8 weeks old, body weight 28.0 ± 1.0 g) were purchased from Viton Lever Laboratory Animal Technology Co., The mice were grouped and individually housed in sterilized room animal cages under a light/dark cycle of 12/12 h, temperature of 22 ± 2°C and humidity of 65 ± 5%. The experimental animals had free access to sterile feed and water, and the bedding used in the cage box was changed once every 3 days. Our protocol ensures the survival welfare of the animals, aims to minimize the number of animals used and the suffering they are subjected to, and was approved by the Ethics Committee for Laboratory Animal Care of Ningbo University (License No. SYXK Zhejiang 2013-0191).

### Grouping and handing of experimental animal

After 1 week of acclimatization to the new environment, the mice were randomly di-vided into 6 groups (15 mice in each group) of control check group (CK), senescence model group (D-gal), positive control group (PC), and sample group (BTE-L, BTE-M, and BTE-H), and treated as follows:

CK group: deionized water (10 mL/Kg) by gavage in the morning and saline (5 mL/Kg) by subcutaneous injection in the afternoon.

D-gal group: deionized water (10 mL/Kg) by gavage in the morning and D-galactose solution (500 mg/Kg) by subcutaneous injection in the afternoon.

PC group: gavage of vitamin C solution (100 mg/Kg) in the morning and subcutaneous injection of D-galactose (500 mg/KG) in the afternoon.

BTE-L group: gavage of BTE low dose solution (100 mg/Kg) in the morning and sub-cutaneous injection of D-galactose (500 mg/Kg) in the afternoon.

BTE-M group: gavage of BTE medium dose solution (200 mg/Kg) in the morning and subcutaneous injection of D-galactose (500 mg/Kg) in the afternoon.

BTE-H group: gavage of BTE high-dose solution (400 mg/Kg) in the morning and subcutaneous injection of D-galactose (500 mg/Kg) in the afternoon.

The experiment lasted for 6 weeks, and the body weights of the mice were recorded each week, and the mice in each group were fasted with food and water for 24 h at the end of the experiment.

### Collection of skin samples

After anesthetizing the mice using 10% chloral hydrate, an electric shaver was used to shave the back of the mice, exposing the skin on the back, and the remaining fine hairs on the skin were removed with a safe depilatory cream and then quickly rinsed with water, wiped dry, and the mice were executed by cervical dislocation. Immediately after execution, 2 × 2 cm of skin was peeled off from the back of the mice, rinsed with pre-cooled saline, subcutaneous fat and connective tissue were removed, and 0.5 × 0.5 cm of the skin was left for determination of skin moisture content. The remaining skin tissues were partly immersed in PBS buffer (0.2 mol/L, pH 7.4) for rapid freezing and preservation for preparation of skin tissue homogenates, and the other part was immersed in 4% paraformaldehyde solution for fixation for 48 h and used for preparation of tissue sections.

### Determination of moisture content in ages skin

The moisture content in the skin was determined by thermostatic drying method by rapidly weighing the mouse skin with a wet weight of 1 g under peeling with a precision instrument, immediately placing it in an oven and drying it at 80°C for 12 h and weighing its dry weight. The moisture content of the skin was calculated according to the following formula:

Moisture content% = (m_1_-m_2_/m_1_) × 100%

m_1_ represents the wet weight of the skin and m_2_ represents the dry weight of the skin.

### Preparation of skin tissue homogenates

Immediately after skin tissue was removed, it was rinsed with iced saline, dry swabbed with filter paper, weighed 0.5 g, cut up, and placed in a centrifuge tube. The measuring cylinder was weighed 4.5 g of pre-cooled PBS buffer (0.2 mol/L, pH 7.4), and the homogenizer was used to make a 10% homogenate of skin tissue, which was centrifuged at 3,600 r/min for 10 min at 4°C, and the supernatant was taken and set aside.

### Measurement of physicochemical indices of aging skin

The prepared skin homogenate was taken, and the standard curve was plotted and operated according to the operating instructions in the manufacturer’s kit. The absorbance (OD value) was measured at 450 nm with an enzyme labeling apparatus, and the contents of HA and MDA, as well as the activities of SOD, CAT, and GSH-Px in mouse skin were calculated from the standard curve.

### Determination of total skin collagen content

Skin tissues were rinsed with ice saline immediately after removal, the filter paper was dry wiped, and 0.5 g was weighed, cut, and placed in a centrifuge tube. The measuring cylinder was weighed 4.5 g of high efficiency RIPA lysate mixed with the skin tissue, and appropriate amount of PMSF solution was added to inhibit proteolysis according to the instructions, and the cells were incubated on ice for 20 min to make the cells fully lysed. The homogenizer was used to make a homogenate of skin tissue, centrifuged at 3,600 r/min at 4°C for 10 min, and the supernatant was taken, and the standard curve was drawn and operated according to the operating instructions in the manufacturer’s kit. Measure the absorbance (OD value) at 450 nm with an enzyme marker, and calculate the content of total collagen in mouse skin by standard curve.

### Preparation of skin sections for HE and immunohistochemical staining

Fixed skin tissues were taken and sequentially immersed in 75% ethanol for 2 h, 85% ethanol for 2 h, 90% ethanol for 2 h, 95% ethanol for 2 h, anhydrous ethanol I for 1 h and anhydrous ethanol II for 1 h for dehydration. The skin tissues were then sequentially immersed in a mixture of xylene and anhydrous ethanol, xylene I, and xylene II for 10 min each for transparency. The skin tissues were then submerged in paraffin for 3 h for embedding, and the tissues were serially sectioned using a microtome. Finally, the slices were spread in warm water at 40°C and placed in an oven at 60°C for 30 min.

### HE staining of skin tissue

The prepared paraffin sections of skin tissue were taken and sequentially immersed in xylene I and xylene II for 20 min each for dewaxing. The sections were then sequentially immersed in anhydrous ethanol I, anhydrous ethanol II, 95% ethanol, 85% ethanol, and 75% ethanol for 5 min each for dehydration, and rinsed with distilled water upon completion. The slices were immersed in hematoxylin staining solution for 5 min, and after completion, they were rinsed with distilled water for 10 min, color separation in 1% hydrochloric acid solution for 10 s, rinsed with distilled water and color separation in 1% ammonia solution for 10 s, rinsed with distilled water, and then immersed in 0.5% eosin solution for 3 min for staining. The sections were sequentially decolorized in 85% ethanol, 95% ethanol, anhydrous ethanol I, and anhydrous ethanol II for 5 min each. The sections were sequentially immersed in xylene I, xylene II, and xylene III for 5 min each until transparent, and the sections were sealed with neutral gum. The sections were examined microscopically under a microscope and the resulting images were analyzed.

### Immunohistochemical staining of skin type I and III collagen

The prepared paraffin sections of skin tissues were taken and sequentially immersed in xylene I and xylene II for 20 min each for decolorization, then put into citric acid (pH 6.0) antigen repair solution and heated in the microwave oven for antigen repair, the microwave oven operation procedure was medium fire for 8 min to boiling, cease fire for 8 min and then turn to medium-low fire for 7 min. After cooling down, slides were washed with PBS (pH 7.4) for 3 times, each time for 5 min.

Endogenous peroxidase was blocked by incubating the sections in 3% hydrogen peroxide solution, protected from light for 25 min. At the end, the slides were washed three times with PBS (pH 7.4) for 5 min each time.

Histochemistry circles were drawn and 3% normal goat serum was added internally dropwise to cover the tissue evenly and closed for 30 min at room temperature.

Sections were placed flat in a wet box, primary antibody was added and incubated overnight at 4°C. After completion, the slides were washed three times with PBS (pH 7.4) for 5 min each time.

The sections were shaken dry, the secondary antibody was added dropwise in the circle to cover the tissue evenly, and incubated at room temperature for 50 min. After completion, the slides were washed three times with PBS (pH 7.4) for 5 min each time.

Shake the section dry, add drops of freshly prepared DAB color-developing solution in the circle, and control the time of color development under the microscope, the positive color is brownish-yellow, and rinse the section with tap water to terminate the color development.

The staining was re-stained using hematoxylin for 3 min, and after completion, the sections were decolorized by placing them sequentially into 85% ethanol, 95% ethanol, anhydrous ethanol I, and anhydrous ethanol II for 5 min each.

The sections were sequentially immersed in xylene I, xylene II, and xylene III for 5 min each until transparent, and the sections were sealed with neutral gum.

Microscopic examination was performed and images were captured and analyzed. The average optical density values of skin type I and III collagen expression were calculated using the ImageJ (v 1.52i) analysis system.

### Statistical analysis

IBM SPSS 22.0 software was selected for data analysis of the experimental data. All data were subjected to at least three repetitions of the experiment, and data information was expressed as mean ± standard deviation, and significant differences between groups were analyzed by one-way analysis of variance (ANOVA). The results obtained were considered statistically significant when *P* < 0.05.

## Results

### Effect of BTE on body weight and food intake in senescent mice

**FIGURE 1:**
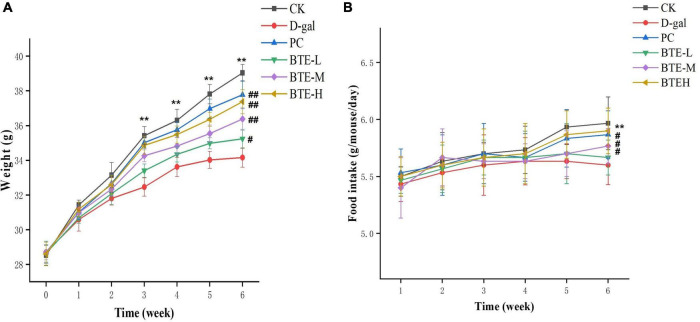
Effect of BTE on body weight **(A)** and food intake **(B)** in aging mice. All data are expressed as mean ± standard deviation of 15 mice per group. ** indicates significant comparisons between the CK and D-gal groups, ** indicates *P* < 0.01; ^#^ indicates significant comparisons between the sample group and the D-gal group, unlabeled indicates *P* > 0.05, # indicates *P* < 0.05, and ^##^ indicates *P* < 0.01. CK, control check group; D-gal, senescence model group; PC, positive control group; BTE-L, BTE low dose solution (100 mg/Kg); BTE-M, BTE medium dose solution (200 mg/Kg); BTE-H, BTE high-dose solution (400 mg/Kg).

The effect of BTE on the body weight of mice is shown in Figure 1A. Before the start of the experiment, there was no significant difference in the body weight of mice between groups (*P* > 0.05). Starting from the 3rd week, the body weight of mice in the CK group was highly significantly higher than that of the D-gal group (*P* < 0.01); at the 6th week of rearing, the body weight of mice in the BTE-L group was significantly different from that of the D-gal group (*P* < 0.05); and the body weights of mice in the PC group, the BTE-M group, and the BTE-H group demonstrated a highly significant difference from that of the D-gal group (*P* < 0.01).

The effect of BTE on the intake of mice is shown in Figure 1B. The weekly recordings of the mice’s food intake revealed that when feeding to the 6th week, there was a highly significant difference between the food intake of the CK group and D-gal mice (*P* < 0.01); there was no significant difference between the food intake of the mice in the BTE-L group and the D-gal group (*P* > 0.05); and there was a significant difference between the food intake of the mice in the PC group, the BTE-M group, and the BTE-H group compared with the D-gal group (*P* < 0.05).

### Effect of BTE on skin moisture and HA content

**FIGURE 2:**
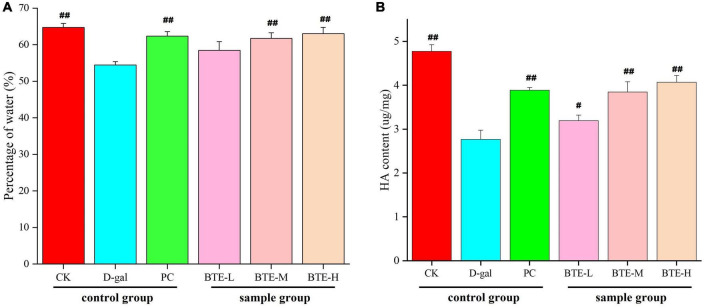
Effect of BTE on skin water **(A)** and HA **(B)** content in aging mice. All data are expressed as mean ± standard deviation of 15 mice per group. ^#^ indicates significant comparisons between each group and the D-gal group, with no markers indicating *P* > 0.05, ^#^ indicating *P* < 0.05, and *^##^* indicating *P* < 0.01. CK, control check group; D-gal, senescence model group; PC, positive control group; BTE-L, BTE low dose solution (100 mg/Kg); BTE-M, BTE medium dose solution (200 mg/Kg); BTE-H, BTE high-dose solution (400 mg/Kg).

The effect of BTE on skin moisture content is shown in Figure 2A. After 6 weeks of feeding, the skin moisture content of both CK and PC groups showed highly significant difference (*P* < 0.01) compared to D-gal group. After BTE intervention, there was no significant difference (*P* > 0.05) in the skin moisture content of the BTE-L group compared to the D-gal group; the BTE-M and BTE-H groups showed highly significant differences (*P* < 0.01) compared to the D-gal group, respectively.

The effect of BTE on skin HA content is shown in Figure 2B. After 6 weeks of feeding, the skin HA content of both CK and PC groups reached a significant difference (*P* < 0.01) compared to the D-gal group. After 6 weeks of BTE intervention, the skin HA content of the BTE-L group showed a significant difference (*P* < 0.05) compared with the D-gal group; both the BTE-M and BTE-H groups reached a highly significant difference (*P* < 0.01).

### Effect of BTE on MDA content in skin

**FIGURE 3:**
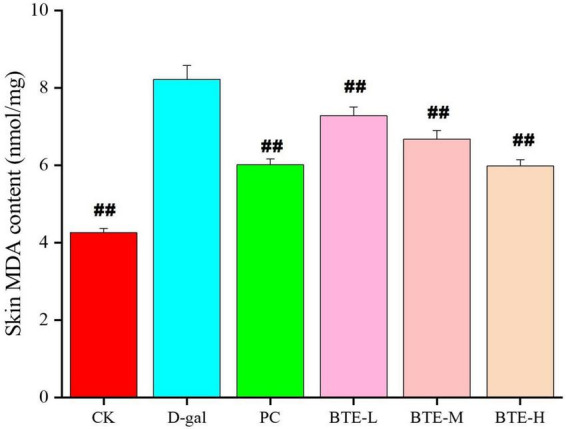
Effect of BTE on MDA content in skin of senescent mice. All data are expressed as mean ± standard deviation of 15 mice per group. ^##^indicates significant comparisons between each group and the D-gal group, with no markers indicating *P* > 0.05, ^##^indicating *P* < 0.05, and indicating *P* < 0.01. CK, control check group; D-gal, senescence model group; PC, positive control group; BTE-L, BTE low dose solution (100 mg/Kg); BTE-M, BTE medium dose solution (200 mg/Kg); BTE-H, BTE high-dose solution (400 mg/Kg).

The effect of BTE on MDA content in the skin is shown in Figure 3. After 6 weeks of feeding, the skin MDA content of mice in the CK and PC groups was significantly lower than that in the D-gal group, and both reached highly significant differences (*P* < 0.01). After BTE intervention, the skin MDA content of mice in the BTE-L, BTE-M, and BTE-H groups all showed significant reductions compared with that of the D-gal group, and the significance analyses all showed highly significant differences (*P* < 0.01).

### Effect of BTE on antioxidant enzyme activity in skin

The effects of BTE on antioxidant enzyme activities in the skin are shown in Table 1. The activities of SOD, CAT, and GSH-Px were significantly lower in the D-gal group compared with the CK group (*P* < 0.01), while there was a significant increase in the PC group compared with the D-gal group, all of which showed highly significant differences (*P* < 0.01).

**TABLE 1:** Effect of BTE on antioxidant enzyme activities in mouse skin.

Groups	Enzyme activity		
	**SOD (U/mg)**	**CAT (U/mg)**	**GSH-Px (U/g)**
CK	37.82 ± 2.83^aa^	74.11 ± 3.93^bb^	811.37 ± 11.92^cc^
D-gal	25.87 ± 2.06	51.84 ± 2.54	587.52 ± 24.46
PC	32.55 ± 2.09^aa^	66.93 ± 1.76^bb^	761.77 ± 42.49^cc^
BTE-L	28.96 ± 2.59	58.85 ± 3.85^b^	628.71 ± 20.66^c^
BTE-M	30.31 ± 1.630^a^	61.81 ± 2.93^bb^	696.24 ± 14.52^cc^
BTE-H	33.34 ± 2.47^aa^	67.95 ± 3.24^bb^	750.66 ± 31.54^cc^

All data are expressed as mean ± standard deviation of 15 mice per group. ^a^, ^b^, and ^c^ indicate significant comparisons with the D-gal group in the same column, and blank labels indicate no significant differences (P > 0.05); ^a^, ^b^, and ^c^ indicate significant differences (P < 0.05); and ^aa^, ^bb^, and ^cc^ indicate highly significant differences (P < 0.01). CK, control check group; D-gal, senescence model group; PC, positive control group; BTE-L, BTE low dose solution (100 mg/Kg); BTE-M, BTE medium dose solution (200 mg/Kg); BTE-H, BTE high-dose solution (400 mg/Kg).

After BTE supplementation, the activity of SOD in the skin increased to some extent in all groups. Compared with the D-gal group, there was no significant difference in the low-dose BTE-L (*P* > 0.05); the medium-dose BTE-M was significantly higher (*P* < 0.05); and the high-dose BTE-H reached a highly significant increase (*P* < 0.01). In terms of CAT activity, compared with the D-gal group, there was a significant improvement in CAT activity within the skin of mice in all groups after BTE intervention, with the BTE-L group reaching a significant difference (*P* < 0.05), and both BTE-M and BTE-H reaching a highly significant difference (*P* < 0.01). In terms of GSH-Px activity, there was a significant increase in GSH-Px activity in the skin of mice in all groups compared to the D-gal group, with BTE-L being a significant difference (*P* < 0.05), and both BTE-M and BTE-H reaching a highly significant difference (*P* < 0.01).

### Pathologic analysis of HE staining of skin tissue

**FIGURE 4:**
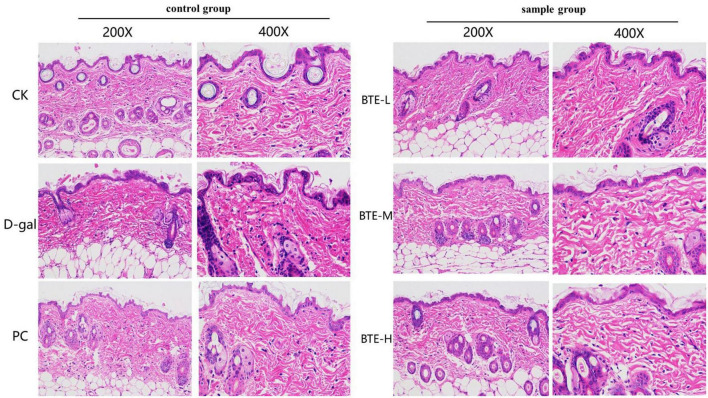
HE staining of aged skin tissue. CK, control check group; D-gal, senescence model group; PC, positive control group; BTE-L, BTE low dose solution (100 mg/Kg); BTE-M, BTE medium dose solution (200 mg/Kg); BTE-H, BTE high-dose solution (400 mg/Kg).

The results of HE staining of senescent skin tissues are shown in Figure 4. In the CK group, the epidermis was intact, the dermis was closely connected to the epidermis, the dermis was thicker, the sebaceous glands and hair follicle cells were tightly arranged, structurally intact, with normal morphology, and the dermal collagen fibers were densely enriched; in the D-gal group, the skin cuticle was atrophied and the dermis was thinned, the hair follicle cells had blurred structures, irregular shapes, the nuclei of the cells were solidly shrunken and aggregated, and the arrangement was chaotic, with the dermal collagen fibers being loosely arranged; in the PC group, the skin dermis was thickened and the tissue morphology was significantly restored; in the BTE-L group, there was no significant difference compared with the D-gal group; in the BTE-M and BTE-H groups, there was a significant improvement compared with the D-gal group, with a better epidermal structural integrity of the mice’s skin tissues, a significant thickening of the dermis, a normal hair follicle cell structure and morphology, and a rich, well-arranged dermis with abundant collagen fibers.

### Measurement of total skin collagen content

**FIGURE 5:**
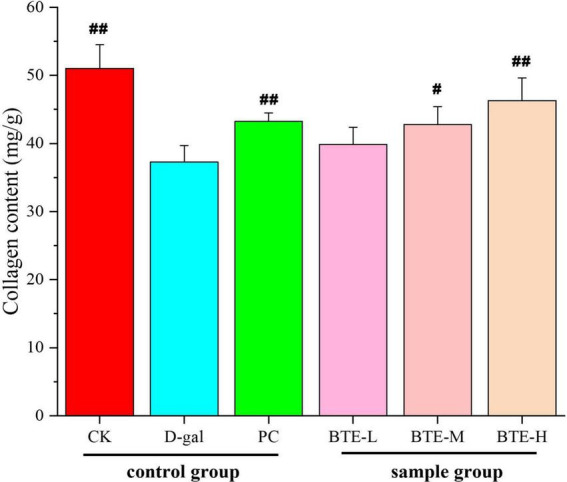
Effect of BTE on skin collagen content of aging mice. All data are expressed as mean ± standard deviation of 15 mice per group. ^#^ indicates significant comparisons between each group and the D-gal group, with no markers indicating *P* > 0.05, ^#^ indicating *P* < 0.05, and ^##^ indicating *P* < 0.01. CK, control check group; D-gal, senescence model group; PC, positive control group; BTE-L, BTE low dose solution (100 mg/Kg); BTE-M, BTE medium dose solution (200 mg/Kg); BTE-H, BTE high-dose solution (400 mg/Kg).

The effect of BTE on the collagen content of aging mouse skin is shown in Figure 5. After 6 weeks of feeding, the collagen content in the skin of mice in the CK group was significantly higher than that in the D-gal group, and there was a highly significant difference between the two groups (*P* < 0.01), and there was a significant rebound in the PC group compared with the D-gal group, and there was a highly significant difference between the two groups (*P* < 0.01). After BTE intervention, collagen loss in the skin of mice was somewhat suppressed at all doses. Among them, the BTE-L group showed less significant improvement in skin collagen content, which was not significantly different from the D-gal group (*P* > 0.05); the BTE-M group showed a greater improvement, which was significant (*P* < 0.05); and the BTE-H group showed a great improvement, which was and significant difference (*P* < 0.01).

### Immunohistochemical staining of skin tissue for type I collagen and type III collagen

**FIGURE 6:**
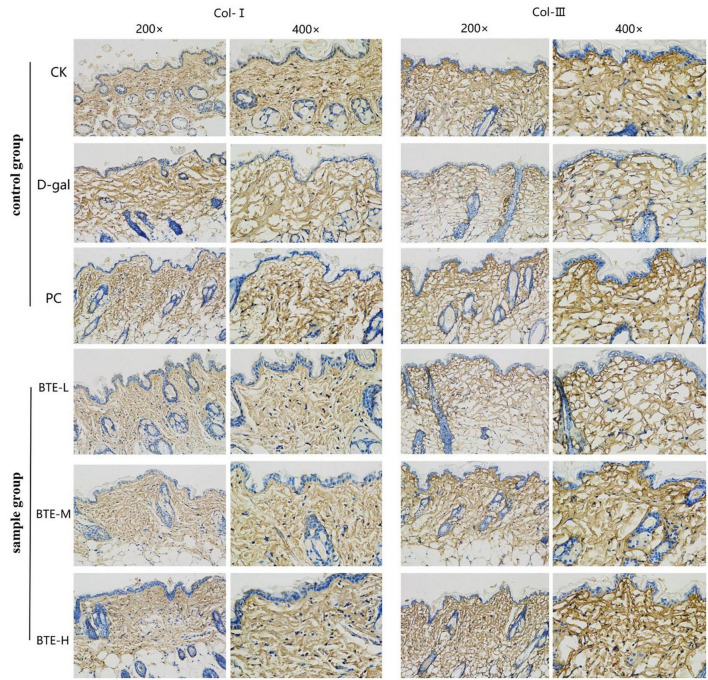
Immunohistochemical staining of aging skin tissue. Positive expression of type I collagen and type III collagen showed brownish yellow color after immunohistochemical staining. CK, control check group; D-gal, senescence model group; PC, positive control group; BTE-L, BTE low dose solution (100 mg/Kg); BTE-M, BTE medium dose solution (200 mg/Kg); BTE-H, BTE high-dose solution (400 mg/Kg).

**FIGURE 7:**
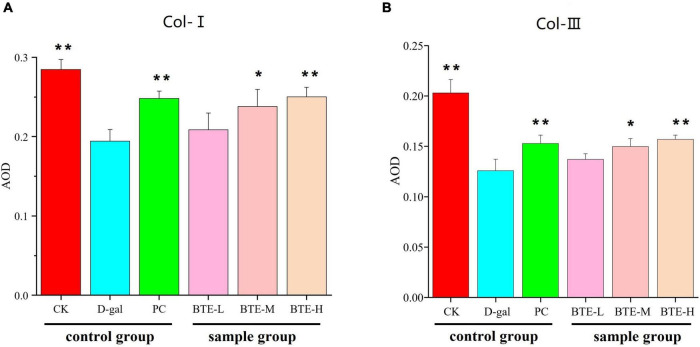
Average optical density values **(A)** Type I collagen **(B)** Type III collagen. All data are expressed as mean ± standard deviation of 15 mice per group. * Indicates significant comparisons between each group and the D-gal group, with no markers indicating *P* > 0.05, * indicating *P* < 0.05, and ** indicating *P* < 0.01. CK, control check group; D-gal, senescence model group; PC: positive control group; BTE-L, BTE low dose solution (100 mg/Kg); BTE-M, BTE medium dose solution (200 mg/Kg); BTE-H, BTE high-dose solution (400 mg/Kg).

Type I collagen (Figure 6 Col-I) and type III collagen (Figure 6 Col-III) were immunohistochemically stained for positive expression showing brownish-yellow color, and the average optical density value (AOD) was calculated using ImageJ software. The mean optical density values of type I collagen are shown in Figure 7A. The expression of type I collagen was significantly lower in the D-gal group compared to the CK group (*P* < 0.01), while the PC group showed a significant improvement compared to the D-gal group (*P* < 0.01). After BTE supplementation, there was no significant enhancement in the BTE-L group compared with D-gal (*P* > 0.05); there was a significant recovery in the BTE-M group (*P* < 0.05); and the expression of type I collagen in BTE-H was highly significantly improved (*P* < 0.01).

The mean optical density values of type III collagen are shown in Figure 7B. Type III collagen expression in the CK and PC groups was significantly higher than that in the D-gal group. After BTE intervention, compared with the D-gal group, the BTE-L group showed no significant improvement (*P* > 0.05); the BTE-M group showed a significant improvement with a significant difference (*P* < 0.05); and the BTE-H group showed a highly significant difference (*P* < 0.01).

## Discussion

The aging of the organism caused by genetic factors is unavoidable (22). Skin, as the largest organ of the human body, is the first barrier to defend against the intrusion of harmful substances from outside, and skin aging is an important feature that reflects the aging of the organism (23, 24). As we age, loss of water, deposition of harmful oxidation products, and decreased activity of antioxidant enzymes in the skin are all important indicators of skin aging, and these changes further accelerate aging (25). Skin aging not only hinders the normal physiological functions, but also has a certain adverse effect on the appearance of the human body, which is manifested in skin atrophy, loss of elasticity and wrinkles (26). Worldwide, the demand for skin care products is expanding and is not limited to cosmetics, as more and more attention is being paid to improving the quality of the skin and slowing down the aging process by modifying the diet.

Black tea contains a variety of bioactive substances that have been shown in past studies to have good anti-inflammatory and antioxidant properties (16, 27, 28). Black tea has a huge consumer base and is one of the most popular teas in the world (15). BTE obtained by hot water extraction retains most of the bioactive substances in black tea and ensures its absolute safety and easy acceptance by consumers (17, 19). Our preliminary experiments determined the content of major phenolics in BTE (Table 2) by high performance liquid chromatography (HPLC). It indicates the active phenolics in BTE have great potential for anti-aging research.

**TABLE 2:** The major phenolic content in BTE.

Ingredient	Content (%)
Theaflavin (TF)	0.41 ± 0.03
Theaflavin 3,3′-digallate (TF-DG)	1.15 ± 0.09
Theaflavin-3-gallate (TF-3-G)	1.39 ± 0.12
Theaflavin-3′-gallate (TF-3’-G)	2.81 ± 0.05
Epigallocatechin (EGC)	1.45 ± 0.07
Catechin (C)	2.81 ± 0.16
Epicatechin (EC)	7.66 ± 0.27
Epigallocatechin gallate (EGCG)	5.89 ± 0.21
Epicatechin gallate (ECG)	4.18 ± 0.14

All data are expressed as mean ± standard.

Weight is an important indicator of the degree of aging of the body. Studies have shown that when an organism grows from childhood to adulthood, its body weight increases rapidly due to its vigorous metabolism and well-functioning organs; however, when it moves from adulthood to old age, due to the deterioration of the gastrointestinal function and weakened metabolism, the elderly individual tends to show a loss of appetite and weight loss (29). Our experimental results showed that D-galactose-induced senescent mice’s body weight and food intake were significantly lower than those of normal mice in the sixth week. Supplementation with BTE effectively reversed this phenomenon, indicating that BTE promoted appetite and restored body weight gain in senescent mice, and that the improvement was positively related to the dose used.

Scientists began exploring the link between moisture content and age as early as the last century. Eventually, they pointed out that the body’s moisture content gradually decreases with age, so the skin’s moisture content has become a key indicator of the level of aging of the skin back in the past (30). Moisture in the skin is primarily found in the dermis. Research in recent years has shown that older people tend to have lower levels of skin moisture than younger people due to the reduction of water channel proteins in the skin and the disruption of normal tissue structure (31, 32). Moisture is a key substance in maintaining the skin barrier, and dehydrated skin is more susceptible to invasion by allergens and microorganisms, triggering skin inflammation. In addition, water loss can cause dryness, loss of elasticity and wrinkles, which can have a significant impact on the appearance of the skin (33). HA, also known as hyaluronic acid, is an acidic mucopolysaccharide, found in large quantities in human skin, joints, eyeballs, and other parts of the human body, because of its unique structure and physicochemical properties in the body plays a variety of important physiological functions (34). In the skin, HA is found mainly in the extracellular matrix layer and is essential for tissue elasticity, hygroscopicity and directly affects skin moisture content (35). Our experimental results showed that BTE intervention increased HA and water content in the skin of aging mice, effectively slowed down the aging process and inhibited skin water loss, and the improvement was proportional to the dose of BTE. In this regard, it is not difficult to infer that daily supplementation of BTE can improve the dryness and flaccidity caused by skin water loss, and maintain the elasticity of the skin.

Malondialdehyde is the main product of the peroxidation reaction of unsaturated fatty acids, which can interact with nucleic acids, proteins, and other biomolecules, causing irreversible damage to the function of the cell, and is extremely cytotoxic (36). MDA is widely present in the human body and accumulates gradually with age, and its large accumulation will lower the body’s immunity and induce skin diseases and various chronic diseases (37). Researchers have detected high levels of MDA in patients with psoriasis and type 2 diabetes, suggesting that MDA is highly pathogenic and poses a threat to human health (38, 39). MDA is often considered a marker of lipid peroxidation in the body to quantify the level of oxidative stress in the organism and is an important indicator of aging (37). The experimental results showed that BTE supplementation significantly reduced the MDA content in the skin of senescent mice, which had a protective and anti-aging effect on the skin. The reduction of MDA content in the skin can prevent the occurrence of skin diseases, which is important for the maintenance of health.

Superoxide dismutase, CAT, and GSH-Px are three important antioxidant enzymes in the human body, which exist in various parts of the body to play the role of antioxidant, forming a protective barrier against free radicals, and the level of their activity is an important weathervane reflecting aging (40, 41). The mechanism of SOD is to catalyze the disproportionation of superoxide anion radicals (O^2–^) to hydrogen peroxide and oxygen, thus scavenging excess free radicals in the body, protecting cells from free radicals, and playing a role in slowing down aging, anti-inflammatory, anti-bacterial, etc., which is now widely used in many fields such as medical and food (42). Past studies have shown that the activity of SOD in the skin of aging individuals is significantly lower than that of younger individuals, and scientists believe that this is one of the main causes of skin lesions and increased symptoms of aging (43, 44). CAT is the hallmark enzyme of the antioxidant enzyme class in the human body, occupying a very high proportion and functioning to catalyze the breakdown of hydrogen peroxide into oxygen and water (45). Hydrogen peroxide can be produced directly by the body’s metabolism, with a certain degree of cytotoxicity, excessive accumulation will stimulate the production of reactive oxygen radicals, resulting in oxidative damage to tissue cells and accelerating the aging of the body (45, 46). Past studies have shown that CAT knockout mice are more susceptible to symptoms of aging compared to normal mice, and the results validate that CAT has a significant anti-aging effect and is widely recognized by the scientific community as a key regulator of cellular aging (47). GSH-Px is an important peroxide-disintegrating enzyme in the organism, which catalyzes the reduction of toxic peroxides to non-toxic hydroxyl compounds, and at the same time, promotes the decomposition of hydrogen peroxide, thus protecting the structure and function of cell membranes from damage caused by peroxides (48). In addition, GSH-Px plays an important role in the maintenance of cardiovascular health, and past studies have revealed its potential in the treatment of cardiovascular diseases, and is an indispensable antioxidant enzyme in the human body (49, 50). According to the results obtained from our study, BTE increased the activities of SOD, CAT, and GSH-Px in aging skin and enhanced the antioxidant defense of the skin, which played a positive role in ameliorating the aging damage of the skin.

The integrity of the skin’s organizational structure is the basic condition for its performance of several biological functions. Current research results show that the skin’s organizational structure can be divided into three parts from top to bottom: the epidermis, the dermis and the subcutaneous tissue (51). The epidermis is located on the outermost part of the skin and can be subdivided into the stratum corneum, stratum pellucidum, stratum granulosum, stratum spinosum, and stratum basale, which plays an important barrier function against external bacteria as well as UV damage (52). The dermis, a thicker layer of fibrous and elastic tissue beneath the epidermis, which can be subdivided into papillary dermis and reticular dermis, provides nutrition and structural support (53). The subcutaneous tissue, which is attached to the dermis, consists mainly of adipocytes and lobules of adipocytes, which have the function of storing energy and relieving mechanical stress (54). Research shows that with the increase of age, the number of fibroblasts in the skin will gradually decrease, and its functional role will also be reduced, manifested in the loss of collagen, the skin loses its original elasticity and toughness, and the tissue structure is inevitably damaged (55). Therefore, maintaining normal skin tissue structure is of great significance in slowing down skin aging. We observed the pathological changes in skin tissues by HE staining and found that the thickness of the dermis layer of the aging skin increased significantly after BTE supplementation, and the integrity of the tissue structure was restored to some extent. The results show that BTE reduces the damage of aging to the skin’s tissue structure and effectively maintains the integrity of the skin’s tissue morphology during the aging process, which ensures that the skin can perform its original biological functions and strengthens the aging skin’s ability to resist external harmful substances. In addition, the restoration of the dermal layer thickness enables the skin to be more elastic and resilient, and to have a better appearance.

Collagen is the most important structural protein in all of the human body and is a component of the extracellular matrix of connective tissues found throughout the skin, cartilage, tendons, and many other organs of the body (56). Collagen is extremely abundant in skin tissues and can account for 50–70% of the total protein content of the skin (56). According to the available information, there are 28 known types of collagen, of which the collagen in skin tissue is mainly type I collagen and type III collagen (57, 58). Collagen is found in the skin primarily in the dermis, a tightly packed mesh of collagen fibers that play a key supporting role in the skin’s tissues and give the skin a certain degree of elasticity and resilience, making the skin look smooth and supple in appearance (59). The amount of collagen in the skin is not fixed and can be lost due to a variety of factors such as age, photodamage, physical trauma, etc. Of these, collagen loss due to aging is the most prevalent and unavoidable (60, 61). The importance of collagen to the structure of the skin has been proven in many ways, and its content in the skin directly reflects the health of the skin. We performed immunohistochemical staining of collagen type I and collagen type III in the skin of aging mice and determined the total collagen content, which showed that BTE reduced the loss of collagen due to aging and effectively protected the structure of skin tissues.

Nowadays, people are taking care of their skin more and more seriously. With the increasing demand, in addition to traditional skin care products, people are gradually turning their attention to diet, hoping to achieve the purpose of slowing down skin aging through the intake of natural bioactive substances. Therefore, the search for safe, effective, and low-cost foods as a means of inhibiting aging is practical and forward-looking. Black tea has a large consumer base in the world, and the safety of its extract has a high guarantee. This experiment confirms that taking BTE has a better effect of anti-skin aging, which provides data support for its subsequent development in this field.

## Conclusion

The results of this experiment reliably showed that oral administration of BTE can effectively increase the content of water, HA, and total collagen in aging skin, enhance the activity of antioxidant enzymes, reduce the accumulation of MDA, and improve skin tissue structure. Among them, the protective effect of medium- and high-dose BTE on the skin was close to or even better than that of vitamin C, indicating that supplementation of the appropriate amount of BTE can effectively delay skin aging and can be used as an anti-skin aging health food. This study provides relevant data for BTE in skin protection, reveals the great market application value of BTE as food in delaying skin aging, and provides some support for the future development of this field.

## Data availability statement

The raw data supporting the conclusions of this article will be made available by the authors, without undue reservation.

## Ethics statement

The animal study was approved by the Ethics Committee for Laboratory Animal Care of Ningbo University (License No. SYXK Zhejiang 2013-0191). The study was conducted in accordance with the local legislation and institutional requirements.

## Author contributions

XZ: Conceptualization, Investigation, Writing-original draft. WD: Validation, Writing-original draft. XW: Methodology, Software, Writing-review and editing. ZW: Software, Writing-review and editing. CL: Resources, Supervision, Writing–review and editing. XZ: Resources, Supervision, Writing–review and editing.
